# Depression in frail older adults: Associations and gender difference

**DOI:** 10.14744/nci.2021.55938

**Published:** 2021-10-26

**Authors:** Firuzan Firat Ozer, Sibel Akin, Tuba Soysal, Bilge Muge Gokcekuyu, Nurdan Senturk Durmus

**Affiliations:** 1 Department of Geriatrics, Kayseri City Hospital, Kayseri, Turkey; 2 Division of Geriatrics, Department of Internal Medicine, Erciyes University Faculty of Medicine, Kayseri, Turkey

**Keywords:** Depression, elderly, frailty, fried criteria

## Abstract

**Objective:**

Depression is highly prevalent in frail older adults and both clinical situations share similar clinical and prognostic factors. The aim of this study was to investigate the relationship between frailty and depression by considering each component of frailty, in both genders.

**Methods:**

Six hundred and forty-one patients aged 60 years and older, admitted to a Geriatrics outpatient clinic in a tertiary hospital, were included in this cross-sectional study. FRIED criteria were used for defining frailty. Depression was assessed by the Geriatric depression scale (GDS). Evaluations related to disability, nutrition, and cognition were performed by the Katz/Lawton scales, mini nutritional assessment (MNA), and the mini mental state examination (MMSE), respectively.

**Results:**

Prevalence of frailty and depression was 48.7% and 36.7%, respectively. Co-occurrence of frailty and depression was observed as 24.0%. Both in men (OR: 3.977, CI: 1.423–11.114, p=0.008) and in women (OR: 2.704, CI: 1.695–4.315, p<0.001), depression was independently associated with frailty, after adjusting with confounders regarding frailty. All FRIED criteria were significantly correlated with the higher GDS scores in women. Two FRIED criteria regarding physical activity and strength measures were not related to depressive symptoms in men. Lawon (p<0.001), MMSE (p=0.004), and MNA scores (p<0.001) were lower in patients with co-occurring frailty and depression, than both as separate conditions.

**Conclusion:**

Depression may play a key role in the development of frailty in both genders. Albeit, depressed men seem to be at a higher risk than women for development of frailty are, physical activity and strength measures in the FRIED criteria had a high impact in women for depressive symptoms. Patients with co-occurring frailty and depression are more prone to develop geriatric syndrome related disabilities.

**B**oth depression and frailty are geriatric syndromes that are highly prevalent in old age but are not expected results of aging [1]. Frailty is the state of vulnerability to stressors due to the reduction of existing biological reserves and is described by many scales, with both phenotype and cumulative deficit models [2, 3]. Furthermore, frailty could explain the great discrepancy, between biological age and chronological age, among the older population [4]. The prevalence of frailty in community-dwelling older adults has been found in a wide range, as 4–59% in cross-sectional studies, depending on the selected method for defining frailty [1]. Depression prevalence has been reported around 10–20%, depending on the elderly population screened [5]. The co-occurrence of depression and frailty has been observed between 16% and 35% [6]. Unintentional weight loss, low physical activity, slowness, decreased muscle strength, and exhaustion, which are the components of the most commonly used tool, the Fried scale, are also frequently encountered clinical conditions in depressed individuals, and exhaustion is an especially common diagnostic criterion for both syndromes [1]. Depression and frailty share similar clinical and prognostic factors [5]. Both are closely related to undesirable consequences such as decreased quality of life, falls, disability, hospitalization, and mortality [7–9]. The studies have shown that there is a bidirectional relationship between frailty and depression [6]. In other words, the incidence of frailty is high in depressed individuals and the incidence of depression is high in frail individuals [6]. In fact, the two syndromes act as both the cause and the result of each other [10]. Moreover, frailty is usually defined with physical phenotypes, and unfortunately social, cognitive, and psychological components of frailty are often overlooked [11].

In this study, we aimed to investigate the relationship between frailty and depression by considering the effects of other confounding factors regarding frailty. We also examined the clinical implications in the state of co-occurrence of frailty and depression. In addition, we investigated whether the relationship between frailty subcomponents and depressive symptoms differs between the genders.

## Materials and Methods

### Patients Included in the Study

This was a cross-sectional study and patients ≥60 years old age, admitted to a Geriatrics outpatient clinic in a tertiary hospital between November 2019 and January 2020, were included in the study. Demographic characteristics of the patients, number of drugs in chronic use, and comorbid diseases were recorded. Patients with acute diseases, active malignancy, restricted mobility, and advanced dementia (Mini mental state examination [MMSE] score <10) were excluded from the study. Patients with ongoing antidepressant therapy were not excluded from the study. Patients, were able to follow instructions and completed the geriatric assessment tests, were included in the study.

### Depression

Depression was evaluated with the Geriatric depression scale (GDS), which has a total of 30 points, with higher scores indicative of more depressive symptoms and a score of 14 and above defined as depression [12].

**Highlight key points**

• Depression was a significant risk factor for being frailty in both genders.
• Although depressed men seem to be at a higher risk than depressed women for developing frailty, two of the frailty components, physical activity and strength measure, have high impact in women.
• Combination of depression and frailty is associated with worse results in terms of functionality, nutritional status, and cognitive functions, than either condition alone.

### Frailty

Frailty was assessed according to the FRIED criteria, including unintentional weight loss, low physical activity, slowness, reduced muscular strength, and exhaustion [2]. More than 5% of total body weight or >4.5 kg loss in the past 12 months was determined as unintentional weight loss. Low physical activity was considered a metabolic equivalent (MET) <600 (MET min/week), according to the short form of the International Physical Activity Questionnaire [13]. Slowness was defined by 4 m gait speed (GS) at a normal pace. Cutoff values were adjusted for height for both genders: For male, GS ≥7 s (height ≤1.73 m) and GS ≥6 s (height >1.73 m) and for female, GS ≥7 s (height ≤1.59 m) and GS ≥6 s (height >1.59 m) were determined as low GS. Muscular strength was measured by a jamar hand held dynamometer. Hand grip strength (HGS) cutoff values were adjusted for body mass index (BMI) for both genders: For male, HGS ≤29 kg (BMI ≤24 kg/m^2^), HGS ≤30 kg (BMI 24.1–26 kg/m^2^), HGS ≤31 kg (BMI 26.1–28 kg/m^2^), and HGS ≤32 kg (BMI >28 kg/m^2^), for female HGS (≤17 kg (BMI ≤23 kg/m^2^), HGS ≤17.3 kg (BMI 23.1–26 kg/m^2^), HGS ≤18 kg (BMI 26.1–29 kg/m^2^), and HGS ≤21 kg (BMI >29 kg/m^2^) were determined as low muscular strength. Exhaustion was described as a negative response to the 21^st^ question (Do you feel full of energy?) of the GDS. Patients meeting at least three criteria were considered to be frail, 1–2 points as pre-frail, and 0 as robust. To be able to perform further analysis, we dichotomized the study population as frail and non-frail (included both pre-frail and robust).

### Other Assessments

Cognitive status was evaluated by the MMSE [14]. Nutritional status was determined by the mini nutritional assessment (MNA) tool [15]. Activities of daily living (ADL) and instrumental ADL (IADL) were assessed by the Katz and Lawton scales, respectively [16, 17]. Lower scores in both scales indicate a more dependent status. Measured serum levels of 25(OH) Vitamin D were recorded. The study was approved by the Erciyes University Ethics Committee of the hospital (Date: February 20, 2019, Decision no: 2019/136) and informed consents were given by all patients.

### Statistical Analysis

A descriptive analysis was performed based on frailty status. The results of the Shapiro–Wilks test, histogram, and q-q plots were examined to assess data normality. A two-sided independent samples t-test, Mann–Whitney U test, and one-way ANOVA test were conducted to compare the differences between continuous variables; while the Pearson Chi-square test or Fisher exact test were used to compare differences between categorical variables. The Bonferroni adjusted Dunn and z tests were applied for multiple comparisons. Pearson or Spearman correlation coefficients were analyzed for evaluating the linear relationship between continuous variables. Crude and adjusted binary logistic regression models were built to examine the risk effect of depression on frailty. Odds ratios (OR) were calculated with 95% confidence intervals (CI). P<0.05 was considered as statistically significant. Analyzes were conducted using SPSS version 22.

## Results

A total of 641 patients (215 M/426 F) were included in the study. Prevalence of frailty and depression was 48.7% and 36.7%, respectively. Older patients (p<0.001) and women (p<0.021) were more frail. Frailty was more prevalent among patients with low education (p<0.001) and without a spouse (p=0.058). A number of used drugs (p=0.027) and comorbid diseases (p=0.041) were higher in frail patients. Nearly, half of the frail patients had depression (p<0.001). GDS scores were higher (p<0.001) and MMSE scores were lower (p<0.001) in frail patients. Clinical characteristics of the patients compared with frailty status are presented in Table 1. Univariate analysis of covariates regarding frailty was performed according to the frailty status in both genders to be able to build multivariable models in both genders separately (Table 2). In multivariable analysis, depression was adjusted for age, education, number of drugs, number of diseases, diabetes mellitus, coronary artery disease, and MMSE scores in women. In men, depression was adjusted for age, BMI, marital status education, smoking, 25(OH)D levels, and MMSE scores. Both in men (OR: 3.977, CI: 1.423–11.114, p=0.008) and in women (OR: 2.704, CI: 1.695–4.315, p<0.001), depression was independently associated with frailty, after adjusting for confounders regarding frailty (Table 3). All subcomponents of frailty were correlated significantly with the higher GDS scores in the total population and in female patients. In male patients, weight loss (p<0.001), exhaustion (p<0.001), and slow GS (p=0.030) were related with the higher GDS scores, while other two FRIED components, strength and low physical activity, were not significantly related with depressive symptoms. Comparison of GDS scores with subcomponents of frailty in both genders is shown in Table 4. Lawton (p<0.001) scores reflecting disability, cognitive status evaluated by the MMSE (p=0.004), and nutritional status determined by MNA scores (p<0.001) were lower in patients with co-occurring frailty and depression, than both either condition alone (Table 5).

**Table 1.** Comparison of demographic and clinical factors according to the frailty status

Variable	Total N=641	Frail N=312	Non-frail N=329	p
Age	72.3±7.4	74.1±7.8	70.6±6.6	<0.001
Sex					0.021
Male	215 (33.5)	92 (29.5)	123 (37.4)	
Female	426 (66.5)	220 (70.5)	206 (62.6)	
BMI kg/m^2^				
Male	28.0±5.3	27.2±5.1	28.6±5.4	0.058
Female	31.0±6.4	31.1±6.3	30.8±6.6	0.708
Marital status (without spouse)	222 (34.4)	117 (37.5)	103 (31.3)	0.058
Education (≤5 years)	542 (84.2)	278 (89.7)	259 (78.7)	<0.001
Current smoker	66 (10.3)	35 (11.3)	29 (8.9)	0.188
Number of drugs	4.1±2.5	4.3±2.6	3.9±2.4	0.027
Number of diseases	2.5±1.4	2.6±1.4	2.3±1.3	0.041
DM	295 (46.0)	133 (42.6)	162 (49.2)	0.055
HT	423 (666.0)	207 (66.6)	216 (65.7)	0.437
CAD	98 (15.3)	54 (17.3)	44 (13.4)	0.104
Depression	235 (36.7)	154 (49.7)	81 (24.8)	<0.001
GDS score	10.0 (5.0–17.0)	13.0 (7.0–20)	7.0 (4–13)	<0.001
MMSE	25.4±4.5	24.3±5.4	26.5±3.3	<0.001
25(OH)vit D	20.5±10.7	20.5±10.7	20.6±10.5	0.877

Mean±SD, median (25^th^-75^th^ percentiles), n (%), BMI: Body mass index; DM: Diabetes mellitus; HT: Hypertension; CAD: Coronary artery diseases; GDS: Geriatric depression scale; MMSE: Minimental state examination.

**Table 2.** Univariate analysis of confounders according to the frailty status in each sex

Variables	Female		Male	
	OR (95%CI)	p	OR (95%CI)	p
Age	1.073 (1.042–1.104)	<0.001	1.078 (1.037–1.121)	<0.001
BMI	1.006 (0.975–1.039)	0.707	0.947 (0.894–1.003)	0.061
Marrital status	1.076 (0.733–1.577)	0.709	1.894 (0.887–4.042)	0.099
Education	2.980 (1.289–6.888)	0.011	1.876 (1.041–3.381)	0.036
Current smoker	1.231 (0.527–2.871)	0.631	1.728 (0.871–3.430)	0.118
Number of drugs	1.096 (1.013–1.186)	0.023	1.028 (0.918–1.150)	0.632
Number of diseases	1.154 (1.003–1.328)	0.045	1.050 (0.874–1.262)	0.605
DM	0.673 (0.460–0.987)	0.042	0.916 (0.525–1.599)	0.757
HT	1.035 (0.672–1.594)	0.875	0.854 (0.497–1.467)	0.568
CAD	1.768 (0.959–3.261)	0.068	1.173 (0.615–2.236)	0.629
Depression	2.832 (1.894–4.234)	<0.001	3.092 (1.648–5.802)	<0.001
MMSE	0.895 (0.851–0.942)	<0.001	0.876 (0.814–0.943)	<0.001
25(OH)vit D	1.012 (0.991–1.032)	0.269	0.968 (0.934–1.003)	0.076

BMI: Body mass index; DM: Diabetes mellitus; HT: Hypertension; CAD: Coronary artery diseases; MMSE: Minimental state examination.

**Table 3.** Relation of depression with frailty in total population and both genders after adjusting by confounders

**Depression**	**Frailty status**			
	**Crude**		**Adjusted**	
	**OR (95%CI)**	**p**	**OR (95%CI)**	**p**
Total	2.986 (2.135–4.177)	<0.001	2.718 (1.769–4.175)	<0.001
Male	3.092 (1.648–5.802)	<0.001	3.977 (1.423–11.114)	0.008
Female	2.832 (1.894–4.234)	<0.001	2.704 (1.695–4.315)	<0.001

Analysis adjusted for age, sex, body mass index, low education, marrital status, number of drugs, number of diseases, DM, CAD and MMSE scores in total population.

**Table 4.** GDS scores in each frailty component in both genders

	Male		Female	
	GDS score	p	GDS score	p
Frailty				
Yes		11.0 (6.0–17.0)	<0.001	14.0 (8.0–21.0)	<0.001
No		6.0 (3.0–10.0)		8.5 (4.0–14.0)
FRIED components				
Weight loss				
Yes	11.0 (6.0–17.0)	<0.001	14.0 (8.5–21.0)	<0.001
No	7.0 (4.0–11.0)		10.0 (5.0–16.0)	
Exhaustion				
Yes	10.0 (6.0–16.5)	<0.001	14.0 (7.50–20.50)	<0.001
No		6.0 (3.0–10.50)		8.0 (4.0–14.0)
Weakness				
Yes		8.0 (4.5–14.0)	0.436	13.0 (7.0–19.50)	0.002
No		7.0 (4.0–14.0)		10.0 (5.0–15.0)
Slow walking				
Yes		10.0 (4.0–16.0)	0.030	13.0 (7.0–20.0)	0.008
No		7.0 (4.0–12.0)		11.0 (5.0–16.0)
Low physical activity				
Yes		8.5 (4.0–14.0)	0.379	13.0 (7.0–19.0)	<0.001
No		7.0 (4.0–13.0)±6.4		7.0 (4.0–13.5)

P<0.05 significant, median (25^th^-75^th^ percentiles); GDS: Geriatric depression scale.

**Table 5.** Comparison of nutritional status, disability and cognitive status among patients with frailty, depression and co-occuring frailty and depression

Variables	Frailty N=158 (24.6%)	Depression N=81 (12.6%)	Frailty and depression N=154 (24.0%)	p
Age	75.2±7.7*	70.1±6.8¶	72.9±7.8*	<0.001
Katz	11.2±1.8	11.5±1.3	10.8±2.2	0.026
Lawton	12.2±5.0*	13.8±3.4*	10.9±5.4¶	<0.001
MNA	22.1±4.0*	22.3±3.4*	19.8±4.3¶	<0.001
MMSE	24.8±4.9	26.1±3.0*	23.8±5.8¶	0.004

P<0.016 significant, significant difference between* and ¶, mean±SD, MNA:Mininütritional assessment; MMSE: Minimental state examination.

## Discussion

In this study, we observed the co-occurrence of frailty and depression as 24.0% and prevalence of depression among frail individuals as 49.4%. Similarly, in the previous studies, concurrent depression was found in 16–35% of frail individuals and the frequency of depression in frail older adults was observed as high as 46.5% [18]. Such a high ratio of co-occurrence of frailty and depression suggests that a mutual cause-effect relationship may be triggered by the same mechanisms of pathways [6, 17], such as cerebrovascular disease, chronic inflammation, oxidative stress, and hypothalamic-pituitary-adrenal axis dysregulation [19]. It has been shown in the previous studies that oxidative stress parameters and inflammation parameters such as C-reactive protein and interleukin-6, which are common pathophysiological mechanisms for both geriatric syndromes, increase in both depressed and frail individuals [20–22]. Moreover, there are some practical explanations for the association between physical frailty and depression in older adults through a common symptomatology. For example, patients with severe depression, tend to be more frail with depression-related lifestyle changes such as physical activity limitation, poor nutritional intake, and non-compliance with treatment, or vice versa, frails may be more prone to depression due to disturbed functional and social activities [1, 23, 24]. Significant association between all subcomponents of the FRIED and depressive symptoms in this study strongly promotes this common symptomatology.

The role of gender in the correlation between depression and frailty has been explored and different results have been observed. Some studies argued that women in depression are more at risk for frailty than men and some concluded otherwise [25–27]. Both frailty and depressive symptoms are more frequent in female gender regardless of the criteria used, since women accumulate more deficit over time and strength is less in women [25]. In this study, both frailty and depression were more prevalent among women (51.6% and 41.6%, respectively). In addition, we observed that depression was independently associated with frailty after adjusting for confounders in both sexes; however, ORs were higher in men than in women, both in crude and adjusted models. In a recent meta-analysis, it was reported that the risk for frailty due to depression was significantly higher in men than in women (pooled OR for men and women: 4.76 and 2.25, respectively). Therefore, older men with depression are more expected to be frail than women with depression. This might be due to different awareness of depression and treatment demands and responses within genders [27].

We observed that all individual frailty criteria were associated with the higher depressive symptoms as shown in the Nordic Research on Ageing Study.[28] Whereas when the data were analyzed considering sex difference, two FRIED criteria other than weight loss, exhaustion, and slow GS were not related to depressive symptoms in male gender, but all criterion continued to significantly associate with depressive symptoms in female patients. Fried criteria defining strength and low physical activity were not significantly correlated with GDS scores in men. However, correlation analysis between the GDS scores and HGS yielded a significant low negative correlation (r=–0.163, p=0.022), while MET values (r=–0.017, p=0.823) were not significantly correlated with GDS scores. In this study, MET values among male patients were relatively low. None of the patients had a MET value above 3× of the suggested minimum physical activity MET value (600 MET). It has been postulated before that the higher levels of physical activity such as moderate intensity activities such as light weight loading, bicycling, or raking in the garden could relieve feelings of depression in men; however, walking could increase feelings of well-being and relieve depressive symptoms in women [29]. Unfortunately, the only physical activity performed by patients in our study population was walking. A minority of patients were doing moderate activity. Yanagita et al. compared the three physical performance measurements, including GS, repeated chair stands, and handgrip strength with depressive symptoms in community-dwelling older men and concluded that only the association of GS and depressive symptoms remained significant after multivariate analysis [30, 31]. However, subgroup analysis in the Nordic Research on Ageing Study showed that mobility disturbance, especially GS, was strongly associated with an increased risk of death in older depressed women but not in depressed men [25]. A general belief is that the increased risk of mortality associated with depression in older age is largely related to accompanying frailty [23].

We observed that co-occurrence of frailty and depression was related with worse scores in scales regarding IADL, nutrition, and cognitive status, than depression or frailty status alone. These results emphasized that there is a vulnerable subgroup of individuals with simultaneous depression and frailty who are predisposed to develop geriatric syndromes such as malnutrition, dependency, and cognitive decline [31]. Furthermore, it was stated previously that the coexistence of frailty and depressive symptoms is also associated with a higher risk of dementia [32].

Strengths of this study are, we evaluated the relationship between frailty and depression by considering the covariates associated with frailty in a broad perspective. In addition, we explored the correlation between frailty and depression in both sexes by examining each sub-component of FRIED scale. The cross-sectional design of the study is the major limitation of this study. Prospective studies enlightening the cause-effect relationship between frailty and depression would be more valuable.

### Conclusion

Depression may play a key role in the development of frailty in both genders. Although depressed men seem to be at a higher risk than depressed women for developing frailty, physical activity and strength measures have a high impact in women [33]. Frailty is a reversible condition that can be treated with assistance in daily living, Vitamin D supplementation, protein enriched diet, and exercise. Rising evidence advocates the benefit of these interventions not only for frailty, but also for the management of depression [10]. The future research is needed in pre-frail and frail individuals, targeting depression and fatigue and investigating how to decrease the degree of frailty status [1]. Whereby depression and frailty frequently co-occur in older adults, these interventions may be especially rewarding for the frail-depressed subgroup [10].
